# Impact of elevated temperature on the physiological and biochemical responses of *Kappaphycus alvarezii* (Rhodophyta)

**DOI:** 10.1371/journal.pone.0239097

**Published:** 2020-09-14

**Authors:** Yushanthini Nair Kumar, Sze-Wan Poong, Claire Gachon, Juliet Brodie, Ahemad Sade, Phaik-Eem Lim

**Affiliations:** 1 Institute of Ocean and Earth Sciences, University of Malaya, Kuala Lumpur, Malaysia; 2 Institute for Advanced Studies, University of Malaya, Kuala Lumpur, Malaysia; 3 Scottish Association for Marine Science, Scottish Marine Institute, Oban, United Kingdom; 4 Unité Molécules de Communication et Adaptation des Micro-organismes, UMR 7245, Muséum National d'Histoire Naturelle, CNRS, Paris, France; 5 Department of Life Sciences, Natural History Museum, London, United Kingdom; 6 Department of Fisheries Sabah, Kota Kinabalu, Sabah, Malaysia; United Arab Emirates University, UNITED ARAB EMIRATES

## Abstract

The eucheumatoids *Kappaphycus* and *Eucheuma* are cultivated in tropical or subtropical regions for the production of carrageenan, a hydrocolloid widely used in the food and cosmetic industries. *Kappaphycus alvarezii* is a highly valued economic crop in the Coral Triangle, with the Philippines, Indonesia and Malaysia ranked among the largest producers. In the absence of measures to mitigate climate change, extreme events including heatwaves, typhoons, severe El Niño and La Niña, are expected to increase in frequency and magnitude. This inadvertently brings adverse effects to the seaweed cultivation industry, especially in the tropics. Temperatures are rapidly reaching the upper limit of biologically tolerable levels and an increase in reports of *ice-ice* and pest outbreaks is attributable to these shifts of environmental parameters. Nevertheless, few reports on the response of eucheumatoids to a changing environment, in particular global warming, are available. Understanding the responses and possible mechanisms for acclimation to warming is crucial for a sustainable seaweed cultivation industry. Here, the physiological and biochemical responses of *K*. *alvarezii* to acute warming indicated that the strain used in the current study is unlikely to survive sudden increases in temperature above 36°C. As temperature increased, the growth rates, photosynthetic performance, phycocolloid quality (carrageenan yield, gel strength and gel viscosity) and pigment content (chlorophyll-*a*, carotenoid and phycobiliproteins) were reduced while the production of reactive oxygen species increased indicating the occurrence of stress in the seaweeds. This study provides a basis for future work on long term acclimation to elevated temperature and mesocosm-based multivariate studies to identify heat-tolerant strains for sustainable cultivation.

## Introduction

Carrageenan, the third most important hydrocolloid in the world after starch and gelatine, occurs as matrix material in *Kappaphycus* [1]. The commercial cultivation of the red alga *Kappaphycus alvarezii* (Doty) Doty ex P.C. Silva has been satisfying the demand for the carrageenan industry over the past four decades by being the biggest source of carrageenan compared to other carrageenophytes such as *Chondrus crispus*, *Hypnea musciformis*, and *Eucheuma denticulatum* [2, 3]. This industrially important carrageenophyte has been mainly farmed for the extraction of carrageenan which is of great economic value in various industries. Realising the potential for commercial extraction of carrageenan, *K*. *alvarezii* was introduced to many countries for research, development, and commercialization by companies. The highly valued economic crop is mainly cultivated in the Philippines, Indonesia and Malaysia, with Indonesia being the leading producer of *K*. *alvarezii* (110,000 MT dry weight), followed by the Philippines with a total production of 60,000 MT dry weight [4].

Carrageenan is a family of gel-forming, viscosifying polysaccharides [5] consisting of sulfated galactans with a backbone of alternating 4-linked α-D- galactopyranosyl and 3-linked β-D-galactopyranosyl monomers. In an effort to improve the economic value, seaweeds are made into Semi Refined Carrageenan (SRC), via a process to meet the quality standards of food (food grade) [6]. The price of seaweed could reach US$ 3.50 to US$ 5.50 per kg once processed into SRC and the value of seaweed carrageenan increases to US$ 9 per kg after Refined Carrageenan (RC) production [7]. Carrageenan is used in a variety of commercial applications as gelling, thickening, and stabilizing agents, especially in the food, cosmetic and pharmaceutical industries [8]. Industrially suitable carrageenan exists in three different forms which are κ-carrageenan (forms strong and rigid gels), ι-carrageenan (forms soft gels) and λ- carrageenan (does not form gels and is used to thicken dairy products) [9]. Among these, κ-carrageenan is highly favoured due to its ability to form stronger gels. It is sourced mainly from *K*. *alvarezii* [10] which is one of the most widely farmed and fast-growing species of *Kappaphycus*.

Cultivation of *K*. *alvarezii* in tropical Southeast Asian countries serves as an important means of livelihood for the coastal communities and a source of income for the countries [11]. Global warming has been an increasing concern and it poses a threat to *K*. *alvarezii* cultivation. According to the Intergovernmental Panel on Climate Change [12], human-induced warming has reached approximately 1°C above pre-industrial levels with a probable range of 0.8 to 1.2°C, and will probably reach 1.5°C between 2030 and 2052 if it continues to increase at the current rate. Temperature rise due to global warming not only causes physical damage to *Kappaphycus* sp. but also affects the eco-physiological, reproductive, and metabolic processes of the seaweed [13]. In Brazil, *K*. *alvarezii* is capable of producing tetraspores, but the reproductive structures fail to complete their germination to successful thallus development due to changes in seawater temperature [14]. Temperature change may cause stress in an organism due to the deleterious effects on enzymatic processes [15]. A study on *Eucheuma denticulatum* showed that the species produced volatile, halogenated, organic compounds when under stress such as from elevated temperature and pH [16].

*Kappaphycus alvarezii* has become more prone to “*ice-ice* disease” due to abrupt changes in temperature of the seawater caused by global warming [8]. The term “*ice-ice*” was developed by farmers in the Philippines to describe the senescent tissue devoid of pigments that caused healthy branches to break off [8]. The occurrence of *ice-ice* has been attributed to abrupt changes in salinity, ocean temperature, and light intensity, which leads to the production of a moist, organic substance that attracts bacteria in the water and induces the characteristic whitening of the seaweed’s tissues [17]. In previous studies, the combined effects of stress and biotic agents, such as opportunistic pathogenic bacteria (for e.g. *Vibrio* sp. and *Cytophaga* sp.) were shown to be primary factors in the development of *ice-ice* disease [15]. Largo *et al*. [13] demonstrated that exposure to water temperatures beyond 30°C could trigger *ice-ice* in *K*. *alvarezii* within one to two weeks. The reported impacts of ice-ice on *K*. *alvarezii* are summarised in Table 1.

**Table 1:** Reported impact of ice-ice on *K*. *alvarezii*.

Components	Impact	References
Carrageenan yield	25–40% reduction	[18]
	Reduced	[19]
Carrageenan viscosity and gel strength	Reduced	[19]
Carrageenan constituents	Lowered levels of iota and methyl constituents	[19]
Photosynthesis	Reduced efficiency	[20, 21]
Photosynthetic pigments	Reduction in concentration leading to reduced photosynthetic efficiency and poor growth	[22]

To the best of our knowledge, reports on the effect of rising temperature on multiple physiological and biochemical responses of *K*. *alvarezii* are yet to be available with most literature focused solely on the impact on photosynthesis [20, 21] nutrient uptake [23] or production of halogenated compounds [16]. Hence, the present study aims to investigate the physiological (growth and photosynthesis) and biochemical (carrageenan yield and quality, pigments and production of reactive oxygen species) responses of *K*. *alvarezii* towards elevated temperature. The results are expected to provide valuable information for farm management practices and to develop strategies to mitigate the effect of global warming, which is imperative for the sustainability of eucheumatoid cultivation and downstream carrageenan processing industries in the tropics.

## Materials and methods

### Ethics statement

*Kappaphycus alvarezii* is not an endangered or protected species. Specimens were purchased from an aquaculture site and were not collected from any national parks or protected areas, thus not requiring any specific permits for sampling.

### Source and acclimatisation of *K*. *alvarezii*

Fresh and visually “healthy” (without showing signs of disease or epi-endophytes and discoloration on the thalli) samples of *K*. *alvarezii* (“Tambalang brown” strain) were obtained from a farm in Semporna, Sabah, Malaysia (GPS coordinate: N 04°29’51.6”, E118°38’36.6”) in April 2019. A set of one-off on-site measurements of the seawater pH, salinity and temperature were obtained as follows: 29±1.0°C, salinity of 30±1.0 ppt and pH of 8.1±0.2 during the fieldwork. The collected samples were transported back to the laboratory and maintained in artificial seawater (Instant Ocean, USA) of 30 ppt in a controlled environment incubator at 28°C with continuous aeration and illuminated with white fluorescent lamps (light and dark cycle of 12h: 12h under the light intensity of approximately 50 μmol photons m^-2^s^-1^) for up to seven days until the maximum quantum yield (*F*_*v*_*/F*_*m*_) reading (one of the photosynthetic parameters) is stable. A representative of the seaweeds was preserved as a voucher specimen [numbered PSM 13033; deposited in the University of Malaya Seaweeds and Seagrasses Herbarium (KLU)] and molecularly confirmed through DNA sequencing with *rbc*L and *cox*1 markers (DNA sequences are available upon request to the corresponding author).

### Temperature experiments

The acclimatised seaweeds were cultured under four different temperatures; 28±1.0 (ambient), 32±1.0, 36±1.0 and 40±1.0°C. Data loggers (Hobo Pendant Wireless Data Logger, Onset Computer Corp.) were used to monitor the temperature fluctuations. The light intensity, photoperiod, pH and salinity for all temperature treatments were as during the acclimatisation. Each temperature treatment was conducted with five replicates and the seaweeds incubated at 28 and 32°C were cultured for fourteen days. An initial biomass of 50±10 g was used for all replicates and temperature treatments. Treatments at 36 and 40°C ended after ten and two days, respectively when the seaweeds were partly or entirely bleached (S1 Fig), thalli became fragile and fragmented and *F*_*v*_*/F*_*m*_ was reduced to ≈0.4 or less. Medium renewal of the artificial seawater was carried out every 2 to 3 days for nutrient replenishment and to overcome evaporation. The growth rate and photosynthetic response were measured every two days, whereas the pigment contents, carrageenan yield and quality were measured on day 0 and on the final day of the temperature treatment (day 14 for 28 and 32°C, day 10 for 36°C and day 2 for 40°C). Production of reactive oxygen species (ROS) was measured on hourly basis (0h, 1h, 2h, 24h and 48h) of the temperature treatments.

### Specific growth rate

The specific growth rate (SGR) was calculated using the equation:

$$\mathrm{Specific}\;\mathrm{growth}\;\mathrm{rate}\;(\%\;{\mathrm{day}}^{-1})=\frac{100\;(\ln\;W_{t}-\ln\;W_{0})}{t}$$

where *W*_0_ = the initial weight and *W*_t_ = the final weight after *t* days.

### Photosynthetic response

Photosynthetic activity of the algal samples was measured by monitoring the chlorophyll fluorescence using a Pulse Amplitude Modulated (PAM) fluorometer (Diving-PAM, Walz, Germany) during the incubation period. The photosynthetic responses were indicated by several parameters including the maximum quantum yield of the sample or

$$\frac{Fv}{Fm}=\frac{\left(Fm–Fo\right)}{Fm}$$

where *F*_*v*_ is the variable fluorescence measured as the difference between maximum (*F*_*m*_) and minimum (*F*_*o*_) fluorescence. Three samples from each replicate were dark-adapted using dark leaf clips for approximately 10 minutes prior to *F*_*v*_*/F*_*m*_ determination. Rapid light curves (RLCs) were generated for all samples using the WinControl software (Walz). The samples were exposed to eight increasing red actinic lights (44, 124, 232, 372, 530, 698, 1001 and 1318 μmol photons m^-2^ s^-1^) for a duration of 10s each to construct RLCs. Other photosynthetic parameters that were measured include maximum relative rates of electron transport rate (rETR_max_), light harvesting efficiency (α) and photoadaptive index (*E*_k_). Values for α and rETR_max_ were calculated by fitting the data from the RLC to an exponential function using a multiple non-linear regression. The intercept of the α value with rETR_max_ gave the saturation irradiance for electron transport (*E*_k_) which is defined [24]

$$\frac{Ek=rETRmax}{\alpha }.$$

### Pigment contents

Chlorophyll-*a* and carotenoids were extracted from approximately 1 g of algal thalli in 10 mL of acetone [25, 26]. For phycobiliproteins analysis, about 1 g of seaweed were ground and extracted in 10 mL of 0.1 M phosphate buffer [25, 26]. The samples were incubated overnight in the dark at 4°C. The homogenate was then centrifuged at 2000 x g for 10 min. Chlorophyll-*a* and carotenoid levels were determined following the equations described by Strickland and Parsons [27] using a UV-Vis spectrophotometer (Shimadzu UV1700, Japan).

$$Chlorophyll‐a\;(\mu g\;g^{‐1})=\frac{A\;x\;volume\;of\;acetone\;(mL)}{fresh\;weight\;of\;sample\;(g)}$$

Where A = 11.6 (Abs_665nm_)– 1.31 (Abs_645nm_)– 0.14 (Abs_630nm_)

$$Carotenoid\;concentration\;(\mu g\;g^{‐1})=\frac{Abs_{452\mathrm{nm}}\;x\;3.86\;x\;volume\;of\;acetone\;(mL)}{Fresh\;weight\;of\;sample\;(g)}$$

Phycobiliprotein levels (phycoerythrin (PE), phycocyanin (PC) and allophycocyanin (APC)) were determined following the equations by Padgett and Krogmann [28].

$$Phycocyanin,\;PC\;(\mathrm{\mu g}\;g^{‐1})=\frac{Abs_{615\mathrm{nm}}\times 0.474(Abs_{652\mathrm{nm}})}{5.34}\times 1000$$

$$Allophycocyanin,\;APC\;(\mu g\;g^{‐1})=\frac{Abs_{652\mathrm{nm}}-0.208(Abs_{615\mathrm{nm}})}{5.09}\times 1000$$

$$Phycoerythrin\;(\mu g\;g^{‐1})=\frac{Abs_{562\mathrm{nm}}-2.41(PC)–0.849(APC)}{9.62}\times 1000$$

Percentage of inhibition was calculated using the formula:

$$Inhibition\;(\%)=\frac{1-X_{\mathrm{final}}}{X_{\mathrm{initial}}}\times 100$$

where *X* are the values of chl-*a*, carotenoid, phycoerythrin, phycocyanin or allophycocyanin.

### Carrageenan extraction

Carrageenan extraction was performed as described by Yong *et al*. [29]. Briefly, seaweeds were rinsed under running tap water and dried in the oven at 60°C overnight. Approximately 4–5 g of the dried seaweeds were ground using liquid nitrogen and the sample weight (SW) was measured. The samples were placed in 50 mL of 70°C pre-heated potassium hydroxide (6% KOH) solution for 30 min. The heated materials were then rinsed with distilled water several times to remove excess KOH before drying in the oven at 60°C overnight to obtain weight (CW) of the semi-refined carrageenan. Carrageenan yield was calculated using the formula:

$$Carrageenan\;yield\;(\%)=\frac{cw}{\mathrm{sw}}\times 100$$

Percentage of inhibition was calculated using the formula:

$$Inhibition\;(\%)=\frac{1-X_{\mathrm{final}}}{X_{\mathrm{initial}}}\times 100$$

where *X* are the values of carrageenan yield.

### Gel strength

The dried semi-refined carrageenan sample (1.5 g) was dissolved in 100 mL of 90°C preheated distilled water to form 1.5% gel solution. The solution was then moulded in small beakers and stored at room temperature prior to analysis (within one to two days). Gel strength was measured using a Nikan-Sui apparatus (Kiya Seisakusho Ltd, Japan). Percentage of inhibition was calculated using the formula:

$$Inhibition\;(\%)=\frac{1-X_{\mathrm{final}}}{X_{\mathrm{initial}}}\times 100$$

where *X* are the values of gel strength.

### Gel viscosity

The dried semi-refined carrageenan sample (1.5 g) was dissolved in 100 mL of 90°C preheated distilled water. Viscosity was determined using the HR3 Discovery rheometer (TA Instrument, USA). The upper heated plate was preheated to 75°C. Spindle rotation was set to 30 rpm. Gel viscosity values were expressed in centripoise (cP). Percentage of inhibition was calculated using the formula:

$$Inhibition\;(\%)=\frac{1-X_{\mathrm{final}}}{X_{\mathrm{initial}}}\times 100$$

where *X* are the values of gel viscosity.

### Reactive oxygen species (ROS) assay

About 0.8 g fresh weight (FW) of seaweed apices were incubated in a solution of 5μM 2’,7’-dichlorofluorescein diacetate (DCFH-DA) (prepared using 10 mM stock solution made in ethanol) in seawater at a density of less than 1 g FW x 100 mL^-1^. Aeration was supplied to the medium to avoid supersaturation of oxygen and to stabilize the pH. The seaweed apices were rinsed with distilled water to remove the excess DCFH-DA and blot dried. The samples were flash frozen in liquid nitrogen and stored at -80°C prior to analysis (within one to two days). The frozen samples were then ground using liquid nitrogen and extracted using 1.25 mL of Tris-HCl buffer (40 mM, pH 7.0), followed by centrifugation for 10 min at 2000 x g at 4°C. The supernatant was diluted 1:2 (v:v) in TrisHCl buffer and used to measure the fluorescence at an excitation wavelength of 488 nm and an emission wavelength of 525 nm using the BioTek Synergy H1 Hybrid Reader (BioTek Instruments Inc., USA).

### Statistical analysis

All data were expressed as mean value ± standard deviation (SD) of five biological replicates. The means of the specific growth rate and photosynthetic parameters were examined for statistical significance (*p*<0.05) between temperature treatments at each time point via one-way analysis of variance (ANOVA) followed by Tukey’s post-hoc test using STATISTICA software (Statsoft, Tulsa, USA). The means of percentage of inhibition in pigment content, carrageenan yield and gel quality were tested for significant differences (*p*<0.05) between temperature treatments using one-way ANOVA and Tukey’s test. The means of absolute DCF fluorescence between temperature treatments and sampling time points were compared for statistical significance (*p* < 0.05) using two-way ANOVA followed by Tukey’s test.

## Results

### Growth rate

**Fig 1:**
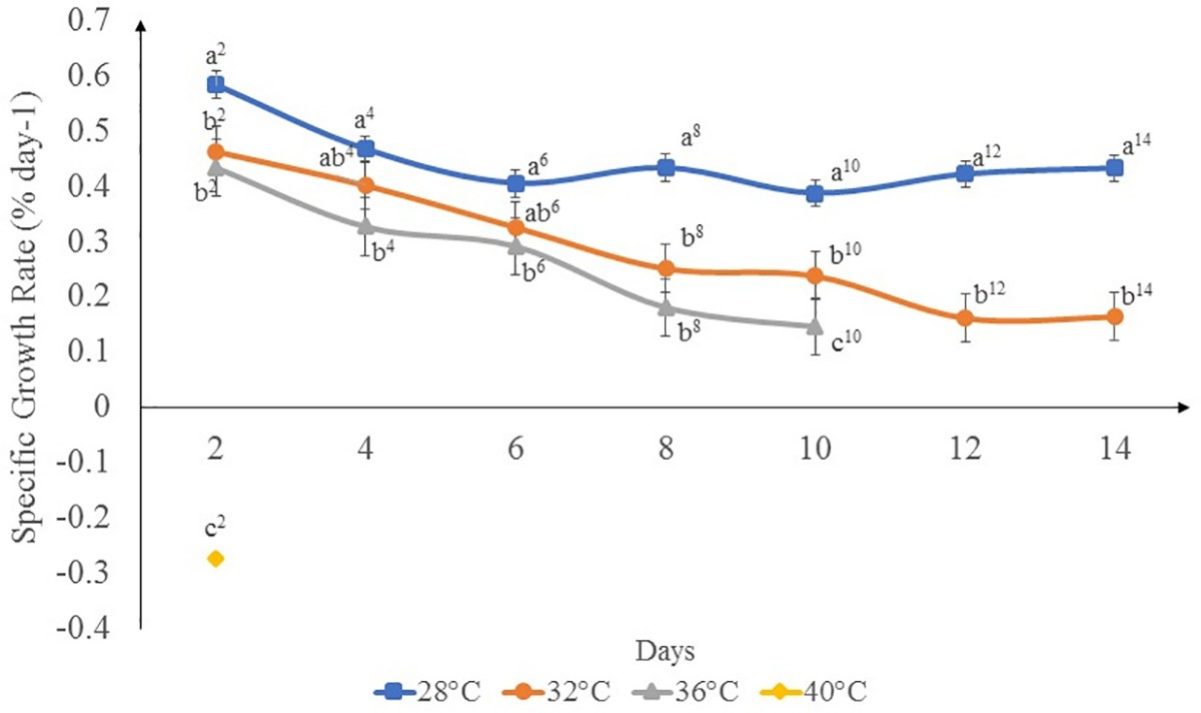
Specific growth rate (% day ^-1^) of *K*. *alvarezii* incubated at different temperatures. Data shown are mean values and standard deviations (*n* = 5). Different lowercase letters indicate significant differences (*p*<0.05) between different temperature treatments at each time point.

The *K*. *alvarezii* strain used in this study demonstrated positive growth rates at temperatures of 28–36°C (Fig 1). Nevertheless, the specific growth rates showed a decreasing trend from day 2 until the end of the experiment. The specimens incubated at 32 and 36°C displayed lower specific growth rates throughout the experiment compared to those incubated at 28°C. Loss of biomass was observed for specimens incubated at 40°C, which indicated that the seaweeds could not survive at this temperature. Significant differences (*p*< 0.05) were observed between the ambient temperature and elevated temperature treatments for each time point. No significant differences (*p*>0.05) were observed between incubations at 32 and 36°C until day 10. Apart from days 4 and 6, growth rate was significantly different (*p*< 0.05) between treatments at 28 and 32°C.

### Photosynthetic response

**Fig 2:**
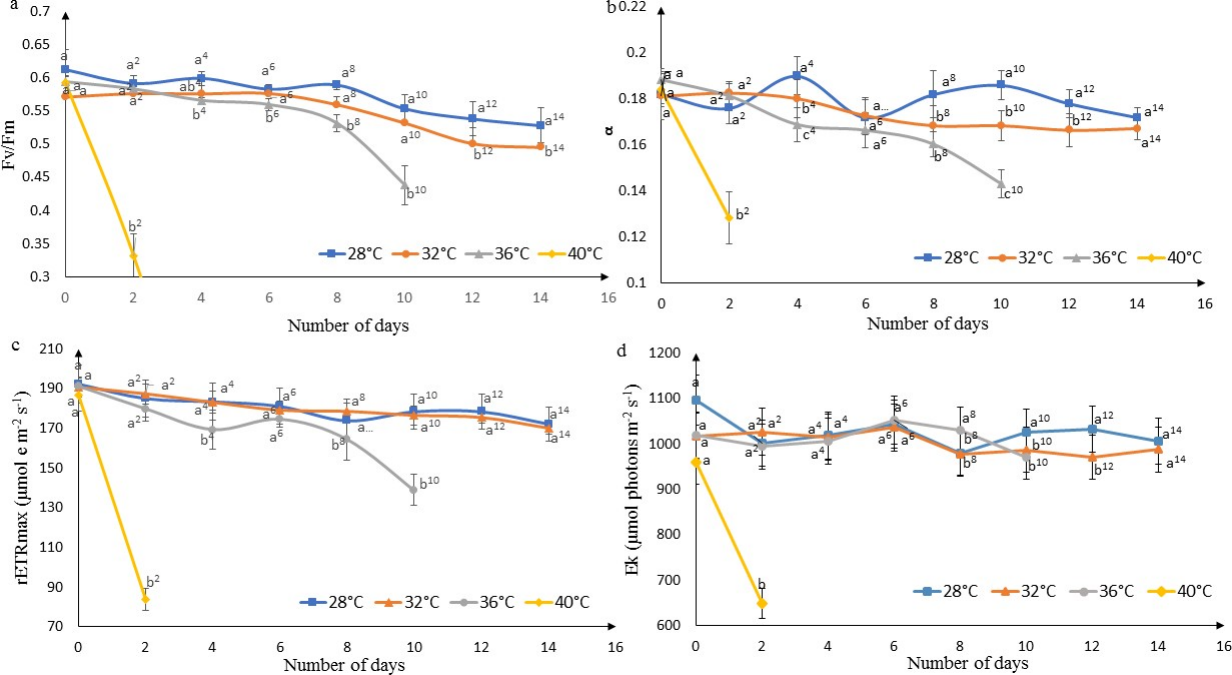
Photosynthetic parameters of *K*. *alvarezii* incubated at different temperatures: a) *F*_*v*_*/F*_*m*_, b) α, c) rETR_max_ and d) *E*_k_. Data shown are mean values and standard deviations (*n* = 5). Different lowercase letters indicate significant differences (*p*<0.05) between different temperature treatments at each time point.

The maximum quantum yield (*F*_*v*_*/F*_*m*_) of *K*. *alvarezii* incubated at 28 to 40°C on day 0 fluctuated between 0.566 to 0.639, displaying the natural variability of *F*_*v*_*/F*_*m*._ and indicating an active photosynthetic state (Fig 2A). The *F*_*v*_*/F*_*m*_ declined noticeably to 0.438 after 10 days of incubation at 36°C. A further increase of temperature (at 40°C) led to a drastic decline to 0.332 ± 0.032 by day 2 and no reading could be obtained on the following day, indicating an impaired physiological state. Maximum quantum yield (or *F*_*v*_*/F*_*m*_) was not significantly different (*p*>0.05) between incubations at 28 and 32°C until day 12 while incubation at 36°C is significant to 28°C and 32°C from day 4 and day 6, respectively. The photosynthetic efficiency, α of *K*. *alvarezii* incubated at 28 and 32°C displayed a similar trend as *F*_*v*_*/F*_*m*_, where the values are stable between 0.170–0.190 throughout the experiment (Fig 2B). *K*. *alvarezii* at 36°C had a slightly higher value of α, (0.188 ± 0.0036) at day 0 but decreased with time to 0.143 ± 0.0059 by day 10, and was significantly different (*p*<0.05) to treatments at 28 and 32°C. Alpha (α) at 40°C was substantially reduced from 0.1838 ± 0.0071 on day 0 to 0.1284 ± 0.0113 by day 2, and significantly different (*p*<0.05) to incubations at 28–32°C. The rETR_max_ of *K*. *alvarezii* incubated at 28 and 32°C remained in the range of 170–190 μmol e m^-2^ s^-1^ throughout the 14 days of incubation (Fig 2C). Incubation at 36°C noticeably inhibited rETR_max_, whereby the magnitude of inhibition increased with incubation time from 191.05 ± 4.62 on day 0 to 138.86±7.81 on day 10. Significant differences (*p*<0.05) were observed between incubations at 36°C and 28°C, and between 36°C and 32°C from day 4 until the end of the experiment with the exception on day 6. The rETR_max_ of *K*. *alvarezii* at 40°C had a sharp decline from 186.64 ± 7.88 on day 0 to 82.80 ± 5.73 after two days of incubation. The saturating irradiance, *E*_k_ of *K*. *alvarezii* incubated at 28, 32 and 36°C fluctuated between 900–1100 μmol photons m^-2^ s^-1^ throughout the experiment (Fig 2D). Incubation at 40°C displayed a significant drop of *E*_k_ from 961.22±43.33 on day 0 to 644.91±102.88 μmol photons m^-2^ s^-1^ on day 2.

### Pigment content

**Fig 3:**
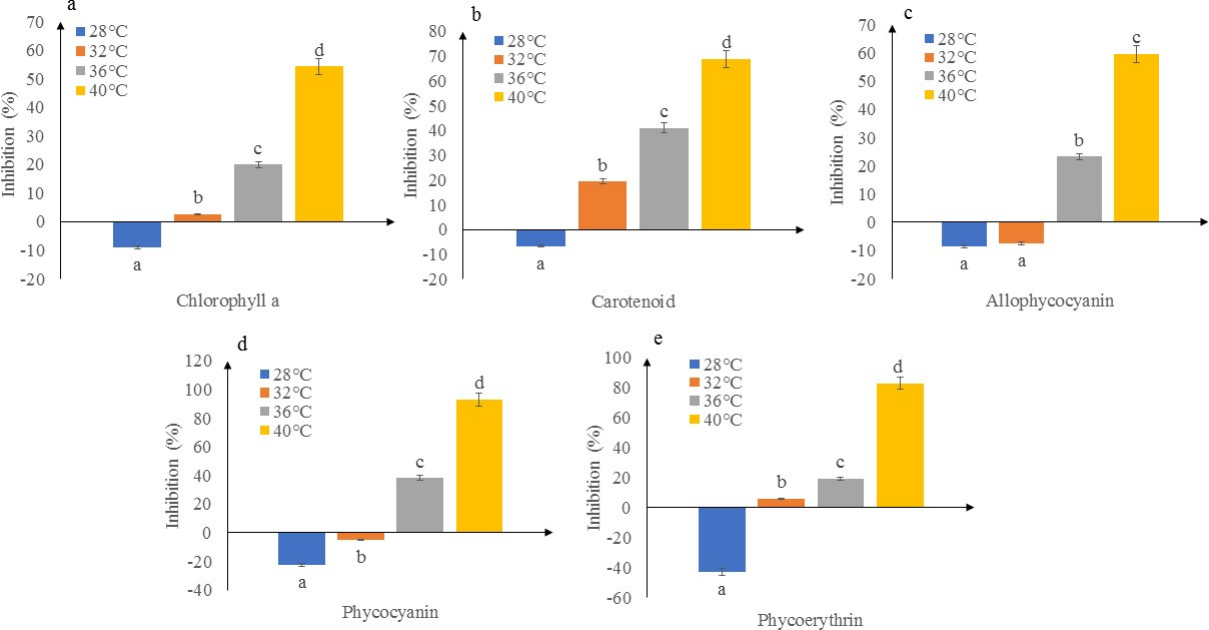
Percentage inhibition of pigment content of *K*. *alvarezii* incubated at different temperatures: a) chlorophyll-*a*, b) carotenoid, c) allophycocyanin, d) phycocyanin and e) phycoerythrin. Data shown are mean values and standard deviations (*n* = 5). Different lowercase letters indicate significant differences (*p*<0.05) between different temperature treatments for each biological parameter.

Incubation of *K*. *alvarezii* beyond the ambient temperature i.e. at 32, 36 and 40°C displayed a reduction in pigment content upon the high temperature treatment (Fig 3, S1 Table). The magnitude of inhibition was positively correlated with the increase in temperature. The percentage of inhibition for chlorophyll-*a* (Fig 3A) was relatively lower compared to carotenoid (Fig 3B), for seaweeds incubated at 32, 36 and 40°C. Significant differences (*p*<0.05) were observed between the four temperatures studied for chlorophyll-*a* and carotenoids. For phycobiliproteins, the percentage of inhibition generally increased with temperature (Fig 3C–3E, S1 Table). The allophycocyanin and phycocyanin content at 28 and 32°C had increased by the end of the experiment. For allophycocyanin, there was no significant difference (*p*>0.05) between the two temperature treatments but for phycoerythrin, only seaweeds incubated at 28°C showed a significant (*p*<0.05) increase by the end of the experiment. The inhibition of phycocyanin and phycoerythrin was significantly different (*p*<0.05) between temperature treatments.

### Carrageenan yield and quality

**Fig 4:**
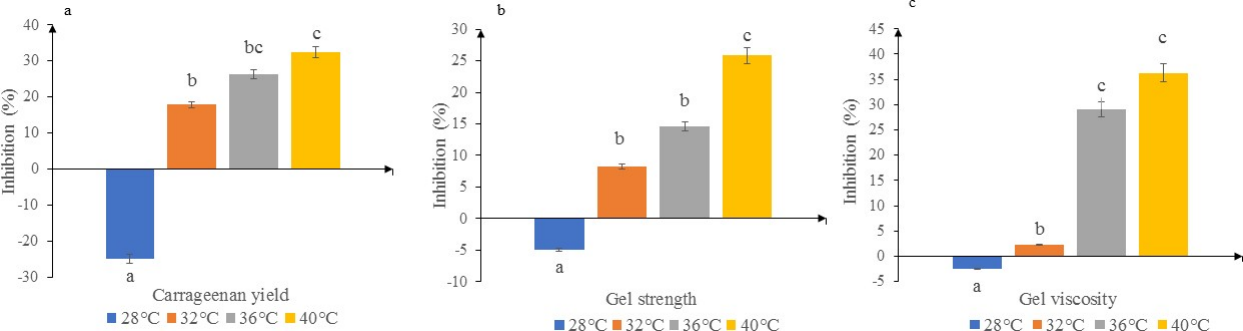
Percentage inhibition of a) carrageenan yield, b) gel strength and c) gel viscosity of *K*. *alvarezii* incubated at different temperatures. Data shown are mean values and standard deviations (*n* = 5). Different lowercase letters indicate significant differences (*p*<0.05) between different temperature treatments for each biological parameter.

The carrageenan yield and quality decreased with increasing temperature (Fig 4, S2 Table) except for 28°C where an increase in yield and quality was observed at the end of the experiment. Significant differences (*p*<0.05) between 32 to 40°C and the ambient temperature were observed for all three biological parameters. No significant differences (*p*>0.05) were observed between i) 32 and 36°C, and ii) 36 and 40°C for carrageenan yield (Fig 4A); between 32 and 36°C for gel strength (Fig 4B); and between 36 and 40°C for gel viscosity.

### Reactive oxygen species (ROS) production

Incubation of the seaweeds at the different temperatures induced the production of ROS which can be assayed via dichlorofluorescein (DCF) fluorescence. The highest ROS production was observed within the first hour and reduced thereafter for all temperatures, in particular for specimens incubated at 36 and 40°C (Table 2). ROS production increased with temperature and the highest values (24941 ± 921) were observed for samples incubated at 40°C. The values fluctuated between 4000–8000 during the following hours, yet the values were temperature dependent, and were generally higher with elevated temperature. Although ROS production started to reduce after the first hour, the values at the 48 h were still relatively higher albeit statistically insignificant (*p*>0.05) than those at 0h for seaweeds incubated at 32, 36 and 40°C. ROS production at 48 h was observed to be slightly lower than those at 0 h for samples incubated at 28°C. Significant difference was observed between 40°C and other temperatures at the 1^st^, 2^nd^ and 24^th^ hour.

**Table 2:** ROS production of *K*. *alvarezii* incubated at different temperatures.

Temperature (°C)	Absolute DCF fluorescense				
	Number of hours				
	0	1	2	24	48
28	4835 ± 782^a^	7871 ± 965^cd^	5794 ± 735 ^ab^	4740 ± 673 ^a^	4201 ± 671^a^
32	4890 ± 391^a^	7702 ± 888^bcd^	5575 ± 464 ^a^	5648 ± 992 ^a^	4928 ± 762^a^
36	4783 ± 530^a^	14197 ± 297^e^	6660 ± 713^abc^	6120 ± 715^abc^	5102 ± 837^a^
40	4826 ± 679 ^a^	24941 ± 921^f^	8276 ± 685 ^d^	8244 ± 581^d^	5298 ± 725 ^a^

Data shown are mean values and standard deviations (*n* = 5). Different lowercase letters indicate significant differences (*p*<0.05) between different temperature treatments and sampling time points.

## Discussion

To the best of our knowledge, this study marks the first report on the effect of elevated temperature on the various physiological and biochemical aspects of *K*. *alvarezii* (Tambalang brown strain) from Malaysia. Our results demonstrate that increasing temperature induced significant impact on the growth rate, photosynthetic performance, carrageenan yield and quality, pigment contents and production of reactive oxygen species (indicative of stress response).

In this study, the growth rate of *K*. *alvarezii* decreased with increasing temperature above 28°C, and the highest growth rate (0.5822% day ^-1^) was recorded at 28°C. The slowing decrease in SGR towards the end of the experiment at 28 and 32°C suggests that the seaweeds may slowly adapt to the environmental changes with time, although this hypothesis needs verification through long-term studies. According to Ohno *et al*. [30], the highest growth rate of *K*. *alvarezii* in Vietnam, was recorded at 25–28°C, whereas the seaweed became fragile with decreased daily growth rates at temperatures of more than 33°C. Similarly, Eswaran *et al*. [31] reported variations in growth pattern of *K*. *alvarezii* in India during different seasons and observed a higher growth rate when the seawater temperature ranged from 26.0 to 28.0°C. Based on these reports, *Kappaphycus* cultivation performs best at 27–30°C in tropical and subtropical waters. Temperature is an essential component which influences the growth rates of *K*. *alvarezii* [32]. In general, the growth and photosynthetic rates of seaweeds increase with temperature until an optimum temperature is reached and then rapidly decline at temperatures above the optimum [33]. Our results showed that the *K*. *alvarezii* strain used in this study can tolerate higher temperatures of up to 32°C fairly well in terms of growth, photosynthetic activity and ROS production, despite reduced pigment content and carrageenan yield and quality by the end of the experiment. Although some seaweeds can adapt to a certain degree of heat stress, prolonged exposure to prolonged high temperature will lead to disruptive stress in the form of cellular and subcellular damage. These damages accompanied by reallocation of resources for protection and repair may lead to slower growth rates [34]. This may be the case in our study whereby the specimens can somewhat tolerate increasing temperatures up to 36°C but only for ten days at most, before the thalli started to bleach and disintegrate. Even at 32°C, significant difference (*p*<0.05) in growth was observed compared to the ambient temperature on day 2 (probably acute stress response to higher temperature) and from day 8 onward (suggesting that the seaweeds start to succumb to heat stress following a brief acclimation period from day 2 until day 6). Crucial metabolic reactions are affected by temperature and thus influence the growth [23]. According to Porter [35], exposure to high temperature above the threshold value (5–10°C above ambient temperature) will cause irreversible damage to the function and development of metabolic cycles.

Shifts in environmental factors potentially cause stress in seaweeds which are often demonstrated in changes to photosynthetic parameters such as *F*_*v*_*/F*_*m*_, α, rETR_max_ and *E*_k_. The maximum quantum yield (*F*_*v*_*/F*_*m*_) is a measure of the efficiency with which photons absorbed by photosystem II (PSII) are used in photochemistry instead of being quenched [36]. Results from our study concurred with previous reports [20, 36] where the *F*_*v*_*/F*_*m*_ is temperature dependent, given that the highest reading is obtained at the optimum temperature (28°C) and reduced at higher temperatures. Among the range of temperatures tested in this study, 28°C appears to be the most favourable temperature for photosynthetic activity of this strain of *K*. *alvarezii*. The effect of temperature on *F*_*v*_*/F*_*m*_ is attributed to the influence of temperature on the oxygen-evolving complex (OEC), enzymatic reactions of carbon fixation, photophosphorylation and electron transport during photosynthesis [20]. The reaction centre of PSII is highly thermolabile and its activity is severely decreased or even inhibited under elevated temperature [37]. High temperature may cause dissociation of the OEC, resulting in an imbalance between the electron flow from OEC toward the acceptor side of PSII in the direction of PSI [38]. Apart from that, the reduction of *F*_*v*_*/F*_*m*_ value with increasing temperature indicates that a structural and functional disorder of the photosynthetic apparatus and damage to the PSII had occurred [39]. Thermal stress produces ROS which inhibits the *de novo* synthesis of D1 protein in PSII, resulting in the decline of *F*_*v*_*/F*_*m*_ [40]. On the other hand, rETR_max_ is defined as the maximum relative electron transport rate and our results indicate that rETR_max_ is greatly inhibited at high temperatures, proving its sensitivity to elevated temperatures (Fig 2C). A similar pattern was observed by Zou and Gao [41] in the marine green macroalga, *Ulva congobata*, where the rETR_max_ values remained stable with moderate fluctuations of temperature (15–30°C) and declined with temperature above 35°C. Our results agree with those by Nishihara *et al*. [21] in which the rETR_max_ of *Eucheuma denticulatum* and *Kappaphycus* sp. (*Sumba* strain) was maximal at 28 to 34°C. The decrease in rETR_max_ beyond the optimal temperature could probably be due to the deactivation of the enzyme rubisco activase that controls the Rubisco activity involved in carbon fixation [42]. Alpha (α) is a measure of the efficiency of carbon fixation per unit light absorbed [43, 44]. Based on our experiment, *K*. *alvarezii* has a higher capacity for photosynthetic activity at 28°C, given the higher values for both rETR_max_ and alpha. The decline of *F*_v_/*F*_m_, rETR_max_ and α at temperatures above 28°C provides evidence of reduced photosynthetic activity due to inhibition of electron transport downstream of PSII. *E*_k_ is the optimum irradiance level for the cells to maintain a balance between photosynthetic energy capture and the capacity of the photosynthetic system to process this energy [45].The higher *E*_k_ values at 28–36°C indicate that the seaweeds were able to acclimatise to elevated temperatures up to 36°C as compared to 40°C. High temperature leads to down-regulation of gene expression in the chloroplast development, resulting in substantial loss of plant photosynthesis which can be attributed to the decline of *E*_k_ and *F*_*v*_*/F*_*m*_ [46].

According to Ganzon-Fortes *et al*. [22], the decline in photosynthesis at elevated temperatures can also be related to pigment damage. A decrease in chlorophyll-*a* content (represented as increase in percentage of inhibition) was observed as temperature increased. This may be attributed to the interference in chlorophyll biosynthesis resulting from inhibition of the electron transport chain [47]. Moreover, high temperature could lead to swelling and dilation of chloroplasts, and fracture of the chloroplast membrane [48]. Impaired chlorophyll biosynthesis and chloroplast development causes reduction in photosynthesis and plant productivity beyond the optimum temperature and this causes the decline in *F*_*v*_*/F*_*m*_, perturbation of thylakoid membrane fluidity and consequent decline in photophosphorylation [49]. The number of main light harvesting pigments (chlorophyll-*a* and carotenoid) determines the ability to absorb light and further affects the growth rate [50], and carotenoid is an accessory pigment which protects chlorophyll-*a* from photodamage [51]. Phycobiliproteins play a crucial role in capturing light energy and transferring it to the chlorophylls during photosynthesis [52]. Phycobiliprotein (allophycocyanin, phycocyanin and phycoerythrin) content was the highest in strains incubated at 28°C and similar findings were observed by Araújo *et al*. [53] for red and brown variants of *K*. *alvarezii*. The direction of energy transfer in phycobilisomes is from phycoerythrin to phycocyanin and to allophycocyanin, which is then eventually transferred to the reaction centre of chlorophyll-*a*. Therefore, external environmental factors such as temperature have adverse effects in these energy transfers and consequently reduce the pigment content [49].

Temperature can affect the yield and quality of carrageenan, as observed in *K*. *alvarezii* (formerly known as *Eucheuma cottonii*) [6]. Our present study, which showed that seaweeds incubated at 32, 36 and 40°C produced significantly lower carrageenan yield compared to seaweeds incubated at 28°C, corroborate with previous studies [53–55], which reported that incubation of seaweeds beyond conducive temperature lowers their gel strength and gel viscosity. According Campo *et al*. [56] the viscosity of the carrageenan is caused by repulsive force in sulphate groups. Consequently, the proportion of sulphate fractions and the equilibrium of cations in the water solution determine the viscosity of solutions or strength of gels formed by carrageenan. The hydrophilic nature of carrageenan causes the water molecule to be intact and causing the carrageenan to be rigid. When seaweeds are exposed to elevated temperature, it causes the loss of the water molecules and subsequently decrease in viscosity and gel strength. This was supported by our observations wherein the percentage inhibition of gel viscosity was higher for seaweeds incubated at 32 to 40°C compared to the ambient temperature. The current data projects catastrophic consequences for the future of *Kappaphycus* cultivation and its relevant stakeholders, given that increased warming may result in reduced carrageenan yield and quality in this economic crop.

ROS is a by-product of metabolism, which includes the superoxide radical and hydroxyl radical [57]. Stepwise reduction of molecular oxygen (O_2_) by high-energy exposure or electron-transfer reactions leads to production of the highly reactive ROS [58]. In plants, ROS are always formed by the inevitable leakage of electrons onto O_2_ from the electron transport activities of chloroplasts, mitochondria, and plasma membranes or as a by-product of various metabolic pathways localized in different cellular compartments [58]. The production of ROS is caused by inhibition of photosynthesis due to suboptimal conditions (e.g. high light, UV, and high or low temperature) or as a secondary response attributable to stress-induced damage [57, 59]. In this study, *K*. *alvarezii* at 40°C exhibited the highest stress-induced production of ROS, which was measured by absolute DCF fluorescence. This is because when seaweeds are exposed to high temperature above the optimum, photoinhibition occurs which leads to accumulation of reduced electron acceptors and may increase the generation of reactive radicals such as ROS which can induce oxidative injuries [60]. Similarly, Ling *et al*. [61] reported that high temperature affects the phytochemical content and antioxidant activity in seaweeds. In this study, the highest ROS production were observed at the first hour for each temperature and reduced thereafter, indicating that the antioxidant activities of *K*. *alvarezii* against the oxidative stress occurred at the beginning of the exposure to elevated temperature and that the seaweeds subsequently experienced thermal adaptation and/or regulated by other defence mechanisms. The increase in magnitude of ROS production with temperature further attests to the severity of stress in seaweeds incubated at higher temperatures.

In conclusion, *K*. *alvarezii* performs best at 28°C among the temperatures tested and signs of stress were observed in *K*. *alvarezii* at 32 and 36°C, while 100% mortality was observed for *K*. *alvarezii* at 40°C within a very short period. Temperatures between 32 and 36°C can be considered as an upper limit temperature for the studied *K*. *alvarezii* as these high temperatures have deleterious effects on the growth and survival of the seaweeds. Temperature is crucial for accelerated growth and increasing photosynthetic performance. However, our study indicated that temperatures of 32°C and above have led to lower pigment content and consequently reduced photosynthetic efficiency, poor growth rates, lower yield and quality of carrageenan and finally *ice-ice* manifestation and loss of biomass due to fragmentation. Thus, for most *K*. *alvarezii*, even small shifts in temperature can have adverse effects on the health of the seaweed, and this would clearly have an impact on the cultivation of this commercially important crop. This study explored the short-term effect of elevated temperature on *K*. *alvarezii* due to technical constraints in maintaining sufficient biomass under laboratory conditions for a prolonged period, but the effect of warming on *Kappaphycus* in the long term remains unknown. Hence, this study provides a basis for future work on long term acclimation to elevated temperatures and is expected to assist the planning and management by various stakeholders in the seaweed aquaculture industry. A projection of how further temperature increase may affect the cultivation of the seaweeds will enable the development of mitigation strategies such as selecting thermal-resilient strains or shifting the timing and/or location of farming, to minimise the risks to the stakeholders’ investments and to ensure a sustainable seaweed cultivation industry.

## Supporting information

S1 Fig Morphological observation of *K. alvarezii* incubated at different temperatures on Day 0, 2, 10 (end of experiment for 36°C treatment) and 14 (end of experiment for 40°C treatment). (TIF)

S1 Table Concentration of pigments extracted from *K. alvarezii* incubated at different temperatures at the start and end of temperature treatments. Values shown are mean ± SD (n = 5).
(DOCX)

S2 Table Carrageenan yield and quality extracted from *K. alvarezii* incubated at different temperatures at the start and end of temperature treatments. Values shown are mean ± SD (*n* = 5).
(DOCX)
