# Supplementary material for: Impact of elevated temperature on the physiological and biochemical responses of Kappaphycus alvarezii (Rhodophyta)
Source: PLoS One. 2020 Sep 14;15(9):e0239097. doi: 10.1371/journal.pone.0239097 (PMC7489555; doi:10.1371/journal.pone.0239097)
Supplement: S1 Table — Values shown are mean ± SD (n = 5). (DOCX) [file pone.0239097.s002.docx]

| Temperature (°C) | Day | Chlorophyll-*a* (µg g^-1^) | Carotenoid (µg g^-1^) | Allophycocyanin (µg g^-1^) | Phycocyanin (µg g^-1^) | Phycoerythrin (µg g^-1^) |
| --- | --- | --- | --- | --- | --- | --- |
| 28 | 0 | 12.184 ± 0.208 | 5.658 ± 0.804 | 4.612 ± 0.718 | 0.329± 0.021 | 4.767 ± 0.781 |
|  | 14 | 13.296 ± 0.539 | 5.929 ± 0.258 | 4.934 ± 0.409 | 0.397± 0.052 | 6.892 ± 0.567 |
| 32 | 0 | 13.573 ± 1.844 | 6.438 ± 1.133 | 4.904 ± 0.460 | 0.322 ± 0.009 | 4.312 ± 0.621 |
|  | 14 | 13.065 ± 1.015 | 5.110 ± 0.544 | 5.231 ± 0.445 | 0.389 ± 0.008 | 4.145 ± 0.601 |
| 36 | 0 | 14.270 ± 1.889 | 6.515 ± 1.151 | 4.55 ± 0.064 | 0.389 ± 0.005 | 4.834 ± 0.178 |
|  | 10 | 11.197 ± 1.774 | 3.643 ± 0.955 | 3.198 ± 0.179 | 0.217 ± 0.010 | 3.891± 0.105 |
| 40 | 0 | 14.188 ± 1.834 | 6.315 ± 1.101 | 5.078 ± 0.218 | 0.365 ± 0.013 | 4.489 ± 0.704 |
|  | 2 | 6.491 ± 2.019 | 2.153 ± 1.207 | 1.877 ± 0.729 | 0.006 ± 0.007 | 0.738 ± 0.563 |
