# Supplementary material for: Impact of elevated temperature on the physiological and biochemical responses of Kappaphycus alvarezii (Rhodophyta)
Source: PLoS One. 2020 Sep 14;15(9):e0239097. doi: 10.1371/journal.pone.0239097 (PMC7489555; doi:10.1371/journal.pone.0239097)
Supplement: S2 Table — Values shown are mean ± SD (n = 5). (DOCX) [file pone.0239097.s003.docx]

| Temperature (°C) | Day | Carrageenan yield (%) | Gel strength (g cm^-2^) | Gel viscosity (cP) |
| --- | --- | --- | --- | --- |
| 28 | 0 | 66.050 ± 4.569 | 548 ± 19.235 | 186.760 ± 2.173 |
|  | 14 | 85.980 ± 2.441 | 580 ± 7.071 | 191.360 ± 4.402 |
| 32 | 0 | 79.159 ± 7.206 | 546 ± 32.093 | 183.740± 7.303 |
|  | 14 | 64.644 ± 2.621 | 500 ± 15.811 | 179.560 ± 4.494 |
| 36 | 0 | 67.396 ± 7.057 | 534 ± 32.093 | 189.440 ± 2.446 |
|  | 10 | 54.792 ± 4.218 | 455 ± 20.00 | 134.220 ± 6.477 |
| 40 | 0 | 73.443 ± 3.581 | 532 ± 28.635 | 187.940 ± 4.061 |
|  | 2 | 49.538 ± 2.936 | 394 ± 11.401 | 119.760 ± 6.881 |
